# Lessons for the global primary care response to COVID-19: a rapid review of evidence from past epidemics

**DOI:** 10.1093/fampra/cmaa142

**Published:** 2021-02-15

**Authors:** Jane Desborough, Sally Hall Dykgraaf, Christine Phillips, Michael Wright, Raglan Maddox, Stephanie Davis, Michael Kidd

**Affiliations:** 1 Department of Health Services Research and Policy, Research School of Population Health, College of Health and Medicine, Australian National University, Canberra Australia; 2 Faculty of Science, Medicine and Health, University of Wollongong, Wollongong, Australia; 3 Australian National University Rural Clinical School, College of Health and Medicine, Australian National University, Canberra, Australia; 4 Australian National University Medical School, College of Health and Medicine Australian National University, Canberra, Australia; 5 Centre for Health Economics Research and Evaluation (CHERE), University Technology Sydney, Sydney, Australia; 6 COVID-19 Primary Care Response Group, Australian Department of Health, Canberra, Australia; 7 National Centre for Epidemiology and Public Health, Research School of Population Health, College of Health and Medicine, Australian National University, Canberra, Australia; 8 Australian Government Department of Health, Canberra, Australia; 9 College of Health and Medicine, Australian National University, Canberra, Australia; 10 Department of Family & Community Medicine, University of Toronto, Toronto, Canada; 11 World Health Organization Collaborating Centre on Family Medicine and Primary Care, Geneva, Switzerland; 12 Murdoch Children’s Research Institute, The Royal Children’s Hospital, Melbourne, Australia; 13 Southgate Institute for Health, Society and Equity, Flinders University, Adelaide, Australia

**Keywords:** COVID-19, epidemics, evidence synthesis, primary care, public health, rapid review

## Abstract

**Background:**

COVID-19 is the fifth and most significant infectious disease epidemic this century. Primary health care providers, which include those working in primary care and public health roles, have critical responsibilities in the management of health emergencies.

**Objective:**

To synthesize accounts of primary care lessons learnt from past epidemics and their relevance to COVID-19.

**Methods:**

We conducted a review of lessons learnt from previous infectious disease epidemics for primary care, and their relevance to COVID-19. We searched PubMed/MEDLINE, PROQUEST and Google Scholar, hand-searched reference lists of included studies, and included research identified through professional contacts.

**Results:**

Of 173 publications identified, 31 publications describing experiences of four epidemics in 11 countries were included. Synthesis of findings identified six key lessons: (i) improve collaboration, communication and integration between public health and primary care; (ii) strengthen the primary health care system; (iii) provide consistent, coordinated and reliable information emanating from a trusted source; (iv) define the role of primary care during pandemics; (v) protect the primary care workforce and the community and (vi) evaluate the effectiveness of interventions.

**Conclusions:**

Evidence highlights distinct challenges to integrating and supporting primary care in response to infectious disease epidemics that have persisted over time, emerging again during COVID-19. These insights provide an opportunity for strengthening, and improved preparedness, that cannot be ignored in a world where the frequency, virility and global reach of infectious disease outbreaks are increasing. It is not too soon to plan for the next pandemic, which may already be on the horizon.

## Teaser

Infectious disease epidemics are increasing in frequency and spread. Primary health care providers are at the forefront of community health care, and have an important role in managing health emergencies such as infectious disease epidemics. It is important to use lessons learnt from past epidemics to inform current health system responses. We examined evidence from past epidemics and identified six key lessons that have emerged in 11 countries during four previous epidemics and again during the COVID-19 pandemic. These lessons highlight the importance of integrating pandemic responses across health care disciplines, strengthening the primary health care system, defining the role of primary care during epidemics, protecting the health care workforce and the community, providing clear and consistent information and evaluating the effectiveness of health care responses. Health system weaknesses are exposed to health emergencies. Identification of these recurring lessons for primary health care provides an opportunity to definitively prepare for future infectious disease epidemics.

**Key messages**

• Primary health care is crucial for infectious disease epidemic management.
• Lessons from the past can improve future health system responses.
• Well-integrated primary care and public health will ensure a cohesive response.
• An effective response requires clear messaging and defined primary care roles.
• A fully functional primary care workforce needs support and protection.
• Evaluation will ensure that epidemic responses are evidence-based and robust.

## Introduction

Several significant infectious disease epidemics have occurred this century. The most notable include Severe Acute Respiratory Syndrome (SARS) during 2002–03 (1); a novel H1N1 influenza A virus in 2009 (2); Middle Eastern Respiratory Syndrome (MERS) in 2012 and 2015 (3); Ebola virus disease (EVD) with 11 outbreaks since 1976 (4), and repeated outbreaks of Zika since 2015 (5). Seventeen years since the SARS crisis another coronavirus, SARS CoV-2, has swept the world. The first reports of the infection were from Wuhan, China in December 2019, with the respiratory disease complex subsequently named COVID-19. At the time of writing (19 November 2020) there have been 56 554 913 cases globally and 1 354 552 deaths (6).

The World Health Organization (WHO) considers that comprehensive primary health care (PHC) is the ‘cornerstone’ of achieving universal health coverage and securing the health of populations around the world (7). PHC encompasses both public health (PH functions and individual patient care, referred to as primary care (PC) (7). While infectious disease epidemics are usually considered through a public health (PH) lens, many of these events have had critical effects on, and implications for, PC settings and providers. Encapsulating these ‘lessons’ in a way that provides clear direction and avoids reiterating past mistakes is of great value during COVID-19 and for future epidemics.

Several reports and research studies have examined the PC response to infectious disease outbreaks and the impact of these on primary care providers (PCP) and communities. However, we were unable to locate any reviews that synthesized accounts of lessons learnt from past epidemics and their relevance to PC. This paper aimed to fill this gap and to determine what can be learnt from previous infectious disease epidemics for PC, and how these lessons are relevant to COVID-19.

## Methods

We conducted a rapid review of the literature, searching PubMed/MEDLINE and PROQUEST without date restrictions for English language material relating to (‘health system response’) AND [‘primary care’ OR ‘primary health care’ OR ‘general practice’ OR ‘family medicine’] AND [SARS OR MERS OR coronavirus OR Zika OR pandemic OR epidemic] AND (AND ‘lessons’ OR “problems). We also searched Google Scholar and hand-searched reference lists of included studies and included grey literature sources known to the authors or identified through professional contacts. We used modified systematic review methods similar to those used in previous rapid reviews (8,9). Speed was a critical concern for the conduct of this review, with the purpose of providing timely and accessible evidence for policy decision-makers. Title and abstract screening were conducted by a single reviewer (SH), with full-text screening and data abstraction undertaken by a separate single reviewer (JD). Systematic assessment of quality or risk of bias was not undertaken for included papers, given the time constraints and the narrative, synthetic nature of many included sources. We acted to reduce the risk of bias in our own analysis through regular discussions contesting and confirming inclusion or exclusion of individual publications to eliminate ambiguity.

### Inclusion and exclusion criteria

We included publications that reflected on experiences during previous pandemics or epidemics and discussed PC lessons learnt or provided PC recommendations for future responses. We included peer-reviewed primary research or commentary as well as reports of reviews or investigations into national responses. We excluded publications reporting hospital or emergency department responses, public health surveillance and descriptive articles about epidemics or COVID-19 responses. While there was no date restriction applied to searches, we noted that no publications before 2001 were identified. We checked the sensitivity of our search strategy by re-conducting searches substituting the MeSH term ‘health systems, international’ for ‘health system response’, identifying earlier publications but none were suitable for inclusion.

### Definitions and conceptual framework

We defined PC as the service domain in which first-contact, ambulatory, biomedical care is provided to individuals in the community, including preventive services, curative and secondary care. PCPs include family doctors (general practitioners and family physicians), nurses, nurse practitioners, community health workers and allied health practitioners. We defined PH as the service domain in which preventive and protective care is provided to communities, including surveillance, monitoring response preparedness, disease prevention, health protection and health promotion (7). PH and PC are conceptualized as allied initiatives which differ in scope and focus, and which have overlapping domains including health promotion, immunization, advocacy for healthy communities and clinical screening (10).

### Data analysis

Each publication was examined and key PC lessons or recommendations arising from the experiences described were identified. These were synthesized and presented thematically. The themes arising from the analysis were discussed at length by two reviewers (JD and SH), with particular attention to the potential for bias.

## Results

**Figure 1:**
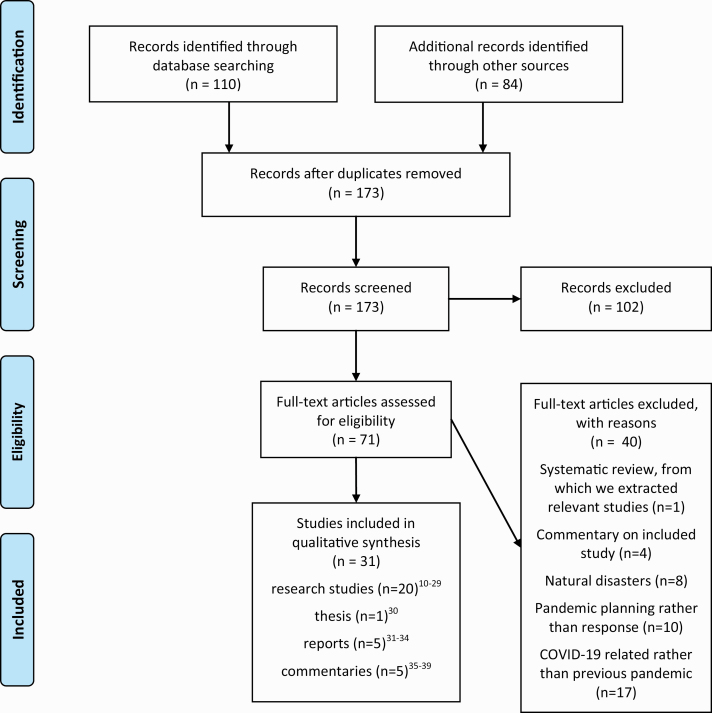
PRISMA flow diagram (adapted from Moher D, Liberati A, Tetzlaff J, Altman DG, The PRISMA Group (2009). Preferred reporting items for systematic reviews and meta-analyses: the PRISMA statement. PLoS Med 6(7): e1000097).

173 articles were identified, of which 31 were included in the review (see Fig. 1: PRISMA Search). These were comprised of 20 research studies (11–30), one thesis (31), five reports (32–36) and five commentaries (37–41). Table 1 summarizes included studies and Table 2 presents countries and epidemics examined.

**Table 1.** Included studies

Citation	Design	Participants	Context	Methods
Austin *et al.*, 2007 (30)	Qualitative	27 pharmacists	SARS, Toronto	Interviews
Herceg *et al.*, 2005 (25)	Quantitative	GPs (*n* = 184) and general practice principals (*n* = 74)	SARS, Australia	Surveys
National Advisory Committee on SARS and Public Health (Naylor Report), 2003 (33)	Mixed methods - third party assessment	46 key informant interviews 31 submissions from public & private organizations/ agencies	SARS, Canada	Source documents Interviews Surveys
Ontario College of Family Physicians, 2003 (34)	Qualitative	Physicians in leadership roles at 2 hospitals, provincially and family physicians.	SARS, Canada	Interviews
SARS Commission: Spring of Fear, 2006 (36)	Mixed methods - third party assessment	SARS Commission – 5-person inquiry team led by Mr Justice Archie Campbell	SARS, Canada	Public hearings − 6 days Public and private submissions Government & hospital documents Interviews (>600)
SARS Expert Committee, 2003 (35)	Mixed methods – expert committee review	SARS Expert Committee	SARS, Hong Kong	Documents and submissions from Department of Health, Hospital Authority, Social Welfare Department, & the public
Tan *et al.*, 2006 (26)	Qualitative	8 GPs	SARS, Singapore	Interviews
Verma *et al.*, 2004 (27)	Quantitative	721 GPs 329 Traditional Chinese Medicine practitioners	SARS, Singapore	Survey
Wong WCW *et al.*, 2004 (12)	Quantitative	183 Clinical Family Medicine tutors	SARS, Hong Kong	Survey
Wong, WCW *et al.*, 2007 (11)	Quantitative	137 PCPs Hong Kong 51 PCPs Toronto	SARS, Hong Kong and Toronto	Survey
Bocquet *et al.*, 2010 (13)	Qualitative	10 General Practice Managers	H1NI, Australia	Interviews
Caley *et al.*, 2010 (23)	Quantitative	367 GPs	H1N1, England	Survey
Eizenberg *et al.*, 2009 (14)	Commentary	PC experiences in northern suburbs of Melbourne, Australia	H1N1, Australia	Opinion
El Emam *et al.*, 2011 (24)	Mixed methods	37 PCPs (5 focus groups & survey)	H1N1, Canada	Focus groups Survey
Grayson & Johnson, 2009 (37)	Commentary	…	H1N1, Australia	Opinion
Kunin *et al.*, 2013 (29)	Qualitative	…	H1N1, Israel, Australia and England	Content analysis of documents released by health authorities during key periods
Kunin *et al.*, 2015 (15)	Qualitative	65 PCPs	H1N1, Israel, Australia and England	Interviews
Lee *et al.*, 2010 (38)	Qualitative	2 PCPs	H1N1, Australia	Personal reflections and synthesis of literature
Masotti *et al.*, 2013 (16)	Qualitative	56 key informants (interviews) 44 PH, PC and government leaders (symposium)	H1N1, Canada	Interviews Symposium
Sweet, 2009 (39)	Qualitative	12 representatives from PC, PH, epidemiology, infectious diseases, consumers, acute care clinicians, & research organizations	H1N1, Australia	Interviews
Tomizuka *et al.*, 2013 (17)	Quantitative	465 PCPs	H1N1, Japan	Survey
Phillips, 2016 (31)	Qualitative	6 GPs	H1N1, Australia	Interviews
Wong SYS *et al.*, 2012 (28)	Mixed methods	300 PCPs (survey)	H1N1, Hong Kong	Survey results extracted
Iyengar *et al.*, 2015 (18)	Quantitative	PHC facilities in Margibi and Bong Counties	EVD, Liberia	Anonymized service utilization data for selected maternal health services at PHC facilities from March to December 2014
Miller *et al.*, 2018 (19)	Qualitative	44 (interviews) 16 focus groups (6–8 participants) with representatives from national and district organizations, and community-level including health care recipients	EVD, Guinea, Liberia and Sierra Leone	Interviews Focus groups
Scott *et al.*, 2016 (41)	Qualitative	…	EVD, West Africa	PHC as a theoretical construct to examine course of pandemic
Siekmans *et al.*, 2017 (20)	Mixed methods	60 CHWs (survey) 16 CHWs (interviews)	EVD, Liberia	Survey Interviews
Wagenaar *et al.*, 2017 (21)	Quantitative	379 public sector health facilities	EVD, Liberia	Time series analysis of 10 key primary health care indicators using 31 836 facility-month service outputs from 1 January 2010 to 31 December 2016
The National Academy of Medicine, 2016 (32)	17-member Commission on a Global Health Risk Framework for the Future	250 invited presenters over 11 days of public meetings in Accra, Ghana; Hong Kong; London; and Washington, DC.	Post-EVD International	Evidence & expert opinion
Al Shehri *et al.*, 2015 (40)	Commentary	…	MERS, Saudi Arabia	Retrospective analysis of experience of MERS in Saudi Arabia
Al-Amri *et al.*, 2019 (22)	Qualitative	85 PCPs	MERS Saudi Arabia	Interviews

**Table 2.** Countries and epidemics examined in included studies

Epidemic	Country
Severe Acute Respiratory Syndrome	Canada (10,29,31,32,34)
	Hong Kong (10,11,33)
	Singapore (25,26)
	Australia (24)
H1N1 Influenza	Australia (12–14,28,30,35–37)
	Canada (15,23)
	England (14,22,28)
	Japan (16)
	Hong Kong (27)
	Israel (14,28)
Ebola Virus Disease	Africa (1)
	Liberia (17–20)
	Guinea and Sierra Leone (18)
	West Africa (39)
Middle Eastern Respiratory Syndrome	Saudi Arabia (21,38)

Six themes and associated sub-themes were identified and are described below. Table 3 provides a matrix of these themes and relevant studies and Table 4 is a thematic summary of studies.

**Table 3.** Matrix of key lessons and studies

	SARS										H1N1														MERS		Ebola					
Lesson	Austin (2007) Canada	Herceg 2005 (Australia)	Naylor Report 2003 (Canada)	Ontario College of Family Practitioners 2003 (Canada)	SARS Commission 2006 (Canada)	SARS Expert Committee 2003 (Hong Kong)	Tan 2006 (Singapore)	Verma 2004 (Singapore)	Wong 2004 (Hong Kong)	Wong 2007 (Hong Kong)	Bocquet 2010 (Australia)	Caley 2010 (England)	Eizenberg 2009 (Australia)	El-Emam 2011 (Canada)		Grayson 2009 (Australia)	Kunin 2013 (Israel, Australia and England)	Kunin 2015 (Israel, Australia and England)	Lee 2010 (Australia)	Masotti 2013 (Canada)	Sweet 2009 (Australia)	Tomizuka 2013 (Japan)	Phillips 2016 (Australia)	Wong 2012 (Hong Kong)	Al Shehri 2015 (Saudi Arabia)	Al Shehri 2019 (Saudi Arabia)	Iyengar 2015 (Liberia)	Miller 2018 (Guinea, Liberia and Sierra Leone)	Scott 2016 (West AFrica)	Siekmans 2017 (Liberia)	Wagenaar 2017 (Liberia)	National Academies of Sciences Engineering Medicine 2016 (Africa)
Collaborate & Communicate (*n* = 18)	✓	✓	✓	✓	✓	✓			✓	✓	✓		✓			✓	✓		✓	✓			✓		✓			✓				✓
Strengthen PC system (*n* = 8)			✓	✓	✓	✓				✓				✓					✓										✓			✓
Provide consistent, coordinated, reliable information (*n* = 17)	✓	✓	✓	✓	✓	✓	✓	✓			✓	✓						✓	✓	✓	✓		✓					✓	✓			
Define PHC role (*n* = 19)		✓			✓	✓		✓	✓	✓	✓						✓	✓	✓	✓	✓	✓	✓	✓			✓	✓	✓	✓	✓	
Protect PHC workforce and public (*n* = 19)	✓	✓	✓	✓	✓	✓	✓	✓	✓				✓			✓			✓	✓	✓	✓	✓	✓		✓		✓				✓
Evaluate (*n* = 2)																			✓									✓				

**Table 4.** Thematic summary of results and studies

Theme/ S*ub-theme*	Epidemic	Lessons/ recommendations
1. Improve collaboration, communication and integration between public health and primary care	SARS	Improve collaboration between government, primary care, public health and hospitals (11,12,30,33–36) Provincial/ Territorial governments need to work closely with local health systems to develop and integrate community pandemic preparedness plans (33) Develop mechanisms to deploy personnel between PC and acute care institutions (11)
	H1N1	Improve collaboration and communication between government, public health and primary care (13,14,16,29,37,38) Ensure appropriate incorporation of primary care in pandemic preparedness plans (14) The public health role of primary care needs to be integrated into public health national plans (29) Improve integration of public health and general practice responses (13) Pandemic response plans need to be contextualized to meet local needs and circumstances, informed by systematic and rigorous consultation with PCPs (15,29,38,39) Engage primary care early and effectively in planning and implementation (increased PH/ PC collaboration), and collaborate with all local stakeholders (16) Local coordination/ national liaison between PC, PHUs and other sectors (38) Local health systems need to work with the PH, PC and the community to develop pandemic preparedness. Need to consider local health networks and PH Unit geographic boundaries (16) Review of public health strategies for communication and workforce protection (13)
	MERS	Improve integration of PC and PH through political and financial support and on the job professional programs for both (40)
	EVD	Engage extensively with communities to build trust (41) Build intersectoral relationships with education, transport, food, labour markets to manage the impact of infectious disease outbreaks on all areas of health and life (19,41)
*Involve PC clinicians in pandemic response planning*	SARS	Involve GPs in pandemic planning (25,34,36)
	H1N1	Increase input into pandemic planning by frontline clinicians, especially general practitioners (37) Direct involvement of primary care clinicians in pandemic planning will recognize and ensure adequate support and protection when undertaking these roles (29) The sense of separation that PC clinicians have from public health officials/ agents (real life/ on the ground) is something that needs to be considered as a barrier to collaboration (31) The commitments and on the ground knowledge of general practices can be harnessed and incorporated into response planning (31)
	EVD	Affected communities need to be treated as essential partners in preparedness and response planning (19) Establish a structure for community-based response prior to the emergency, and then engage again with community actors early in the emergency response (19)
*Improve everyday communication between PH, PC and the rest of the health system*	SARS	Improve everyday communication between family physicians and the rest of the health system as a foundation for crucial involvement in outbreaks (33,34) Improve day to day communication between public health officials and clinicians, including pharmacists (30) and primary care doctors (33,35) Improve communication within and between health agencies (25) Review public health strategies for communication and workforce protection (13)
2. Strengthen the primary health care system	SARS	A shortfall in ambulatory care capacity in a system that relied almost solely on ED resulted in a call for PC reform (33) Address human resource shortages in both sectors (primary and tertiary) (34) Increase the number of family doctors, and attract students and residents (34)
	EVD	Investment in primary health and public health systems to strengthen response to infectious diseases as well as the core capabilities of these systems. These include the management of endemic infectious diseases, which will be strengthened through enhanced surveillance and response systems, and non-infectious diseases (32) Develop a strong primary and community care system, which includes building a strong network of community health workers and PC facilities, including in remote areas (41) Need for formal recognition of traditional birth attendants (TBAs) and community health committee (CHC) members, including development of a role in planning, implementing, financing and monitoring community health initiatives (19) Provide adequate support for community health workers, including paid salaries and motivators such as opportunities for scholarships to support career development, preferential selection to work on health campaigns, education for children, micro-financing for small businesses (19) Find solutions to the financing, scale up and institutionalization of community health services, including increased government funding (19)
*Support PC services in their key role as sentinel systems*	SARS	Assembly of electronic surveillance data for a range of providers, including PC (33,36) Develop an active and collaborative disease surveillance system that integrates frontline and public health systems (11) Extend the sentinel surveillance system in PC (35) Support PC doctors’ role in situation monitoring and assessment – case reporting and monitoring (38) Support family doctors in their key role of sentinels through the provision of an early warning system that can be activated when needed and enable coordination between PC, emergency departments and assessment clinics (34) Create and coordinate real-time alert systems that extend to all health care facilities, including PC (33,36) Assign public health nurses to family care offices to support health promotion, disease prevention and surveillance activities (34)
	H1N1	Address clinician’s concerns about patient privacy to optimize comfort with data sharing (24)
	EVD	Parallel strengthening of primary health care and public health will enable planning and mobilization of a response at the scale required, and will enable sentinel case detection and a health system equipped to respond. Public health and PC both have capacity to act as sentinels (32) Development of a strong network of CHWs and PC facilities will enhance the sentinel role in the community (41)
3. Provide consistent, coordinated and reliable information emanating from a trusted source	SARS	One source of consistent and accurate advice (34,35) Provide timely information and, detailed guidelines and protocols for general practice (25,26,36) and pharmacies (30) Providing accurate, prompt and transparent guidelines and information updates supports psychological needs of HCWs (27)
	H1N1	Provide consistent, accurate information from one single authoritative source (16,23,39) Collaborate to develop consistent messages (16) Reduce duplication of information and increase clarity (23) Need to develop specific guidelines for First Nations people (16) Designate the role of information coordinator (13) Provide multidisciplinary messaging, with linkages between clinical groups such as obstetricians, gynaecologists, GPs and infectious diseases experts and public health (39) Improve communication within and between health agencies (25)
	EVD	Provision of clear and consistent guidance to CHWs and other community actors; with roles, responsibilities and lines of reporting delineated during the planning phase (19) Provide information and awareness for the public to understand why HCWs use PPE (26) The government should provide more public education during an infectious disease outbreak (25)
*Provide clear and consistent guidance for the community*	SARS	GPs are in a strong position to unpack some of the fear about a pandemic for their patients, while encouraging them to prepare realistically (31)
	H1N1	GPs are well placed to communicate with, role model and educate patients and the community regarding infection control and reducing disease spread (38) In order to meet the needs of the public need for information from a trusted source, PC providers need to receive information from health authorities ahead of the media (15) The provision of targeted messaging for distribution in general practice to the public would described some of the statistics that they see in the media and assist in allaying some fears (31) GPs are a local community resource, and can also feed community concerns back to national authorities (38)
	EVD	Establish a network of community health workers embedded in communities prior to disease outbreaks, to ensure established relationships that can be relied on during emergencies (41)
4. Define the role of PC during pandemics	SARS & H1N1	PC physicians need to be fully informed from the outset of their roles and the support they will be provided to implement the response (16,29,30,35,36)
*PC clinicians need to* *be aware of pandemic preparedness plans*	H1N1	PC clinicians need to have access to a PC action plan prior to a disease outbreak. All staff - administrative and clinical need to understand their roles in these plans (38) PC clinicians need to be aware of and familiar with pandemic guidelines in order to conform (15) Implementation of business continuity plans is best supported through familiarity with the national pandemic preparedness plan (17) Leadership within general practice is important, in particular in relation to ensuring practice plans are in place and implemented and identifying and supporting vulnerable patients and staff (39) Clinics should assess practice capacity to perform both PH and PC roles, including hygiene protocols and disaster plans, and a ’flu champion’ (13)
*Provide pandemic preparedness training*	SARS	Provide staff education via face-to-face workshops (25)
	H1N1	Opportunities to test pandemic plans can lead to improved links between policy makers and clinicians (37)
*Provide PC-tailored infection prevention and control training*	SARS	Develop guidelines for infection prevention and control for PC (12) Provide timely and relevant information and training for PCPs regarding the infectious disease outbreak and associated requirement for use of PPE (26,30,34) Incorporate infection prevention and control training for PC practitioners and staff of residential aged care facilities (35) Include infection prevention and control training in ongoing medical education (11)
	H1N1	Elucidate the education needs of PC providers and provide relevant education about how to deal with the infectious disease outbreak (28) Infection prevention and control guidelines need to consider the infrastructure and resource capacity of PC, including the importance of personalized communication between clinicians and patients (15) Explore the reasons for a lack of handwashing amongst doctors and find ways to reduce this risky behaviour (28)
	MERS	Increase education about handwashing among frontline doctors (28)
	SARS	Increased availability of infectious disease education and training and find ways to increase attendance (22)
*Clarify pathways of care for potential and diagnosed cases, and maintain access to regular health care*	H1N1	Set up screening stations away from clinics, and suspected or diagnosed patients sent straight to hospital for treatment (27) Use of centralized assessment centres (25) Planning to enable effective segregation of suspected cases from others (15) Set up designated influenza assessment centres (13,16)
	EVD	Reduce spread of disease - maintain regular health care alongside flu-like illnesses, home care when possible, management of stable patients released early from hospital (38) Divert influenza like illnesses to ensure maintenance of routine care, e.g. assign worried well to a nurse (13) It is essential to consider the unintended consequences of putting aside usual care during disease outbreaks (21) The consequences of a pandemic on all aspects of health care need to be considered, so as to avoid preventable morbidity and mortality (18) Continuation of proven effective health interventions needs to be considered to mitigate poor outcomes for babies, mothers and families (18) Trusted community based health workers are essential in providing continued access to PC are essential during a pandemic (20)
5. Protect the PC workforce and the community *Promote health protective behaviours, psychological wellbeing and business continuity*	SARS	Provide government support for PC doctors in the form of financial support and a centrally organized contingency plan to mitigate their exposure to risks during infectious disease outbreaks (12) The psychological wellbeing of HCWs is supported through clarity of information and guidelines, pathways of care and availability of personal protective equipment PPE (27,34) Support PC clinicians to work to their best to avoid unwarranted psychological stress (16) Need for simple and timely approval processes for laboratory tests and rapid provision of results (13)
*Identify vulnerable and at risk groups*	H1N1	Identify vulnerable and at risk patients and staff for health protection (38,39)
*Ensure access to personal protective equipment (PPE)*	SARS	Ensure access to adequate supplies of PPE to PC (27,33–35) Reliable sources of supplies and equipment required (34) Provide financial and practical support for family doctors to obtain PPE (25,26)
	H1N1	Ensure access to adequate supplies of PPE to PC (31,39), particularly for vulnerable staff and patients (39) Transparency of the size of the PPE stockpile and process for distribution, including development of a mechanism to ensure ready release of PPE to general practices in the states and territories (14)
*Ensure access to antiviral treatments and influenza vaccines*	SARS	Provide staff vaccinations (25)
	H1N1	Address the need for personal and family protection of HCP during a pandemic (16) Need for availability of antiviral therapies as requested, in particular before staff become symptomatic (14) Ensure adequate access to antivirals, particularly for vulnerable staff and patients (39)
*Provide clear guidance for use of antiviral treatments*	H1N1	Guidelines for use of antiviral therapy would be improved if they were adjusted to consider severity of cases (15) Provide guidelines to ensure that PC clinicians are familiar with antiviral therapies (15) The provision of clear guidelines to alleviate GPs’ burden of having to make decisions/ choices when it came to the provision of antivirals and possibly other treatments (31)
6. Evaluate the effectiveness of interventions	H1N1	Conduct clinical audits to assess structure, process and outcomes of PC action plans (38) Determine effective interventions to increase uptake of the influenza vaccine amongst HCWs (28)
	EVD	Routine, rigorous assessment of the program should be included in countries’ monitoring and evaluation plans (e.g. training, supervision, drug supplies, accessibility) (19)



#### Improve collaboration, communication and integration between public health and primary care

Eighteen studies highlighted the need to improve collaboration and day-to-day communication between PH and PC (11–13,30,32–36,38,40), between PC and hospitals (28,35), between different levels of government (11,16,38) and between public and private sectors (including PC) (11,12). PCPs’ experiences pointed to a focus on hospital care at the expense of PC services which were provided with inadequate information and support (30,34,36), and failures to formally engage stakeholders at the forefront of providing community-level care, including PCPs (33,34,36), pharmacists (30), birth attendants and traditional healers (19). This included a need for direct lines of communication from health care workers to PH (34,36).

##### Include PCPs in pandemic preparedness plans

Inclusion of PCPs in pandemic response planning was advocated to recognize the essential role of PC in service delivery for communities, to contextualize responses to local circumstances and ensure adequate support and protection (15,19,25,29,34,37–41). Local integration of preparedness planning across sectors (government, local health systems, PH units, PCPs) was recommended, focused on community need and supported by political, financial and educational inputs (16,33,36). Barriers to collaboration between PC and PH included a shared misunderstanding by each of the functions and responsibilities of the other discipline (31), and a mismatch between the two disciplines’ roles in terms of authority and responsibility (16)

#### Strengthen the primary health care system

Eight studies recommended strengthening the PC system in tandem with PH capability (11,24,32–35,38,41), in advance of, rather than in parallel with, disease outbreaks (33,34,41). Reviews of the SARS experience in Canada (33,34,36) and Hong Kong (11) noted shortfalls in PC capacity, resulting in increased reliance on emergency departments and community pharmacies (30) that were also experiencing human resource shortages. Recommendations included increasing PCP numbers, attracting medical students and residents to primary care (34) and deploying personnel between institutions as surge support during epidemics (11). Investment in strong primary and community care systems with embedded networks of health workers was recommended to provide a trusted source of community engagement that could be relied upon during emergencies (32), and offer care for those who may be otherwise reluctant to seek treatment (41). This would require solutions to the financing, scale-up and institutionalization of community health services (19).

##### Support PHC services as sentinel systems

Expert reviews of responses to SARS in Canada and Hong Kong, referred to a need to support the key sentinel role of primary care (34–36), through the assembly of electronic surveillance data (33) and extending existing surveillance systems (11,35). Researchers argued that improved collaboration between PC, PH and other providers would contribute to the provision and coordination of real-time alert systems for managing infectious diseases (11,33,34), and enable planning and mobilization at the required scale, enhancing the sentinel role of both (32).

#### Provide consistent, coordinated and reliable information emanating from a trusted source

Experiences from SARS (25–27,33–35), H1N1 (13,16,25,29,31,34,39,42) and EVD (19,41) resulted in calls for provision of consistent and reliable information, distributed by a trusted source (13,16,23,34,35,39). PCPs described multiple pieces of information coming from many, often conflicting, sources, a lack of PC-tailored information and no established route for providing feedback about policies (15,25,31). Recommendations were for a reduction in duplication, increased clarity of communication (23) and provision of clear, consistent guidance (19). There was a lack of guidelines focusing on rural or remote populations, or the needs of First Nations people (16). At times, opinions differed between infectious disease experts about risks and implications of emerging evidence, making coherence difficult to achieve (39).

Community members also grappled with inconsistent messaging regarding risk and protective behaviours, resulting in fear and sometimes unwarranted presentation to health services (39). Targeted community messaging from PC was seen as one way to allay public anxiety and support the factual interpretation of media reports (26,31).

#### Define the role of primary care during pandemics

The need for PC role definition was referred to in studies examining SARS (11,12,25,35,36), H1N1 (13,15–17,28,29,31,38,39) and EVD (19). Lack of role clarity was a source of distress for PCPs, including pharmacists (30) during SARS outbreaks in Ontario (34,36) and Hong Kong (35). Following H1N1, Canadian PCPs described personal trauma related to lack of clarity in pandemic influenza plans (PIPs), and planning and logistical issues, whereas staff working in centres with more detailed PIPs reported less stress and fewer unforeseen problems and delays (16). Australian PCPs perceived conflict between their PH role and usual clinical care responsibilities, largely driven by a lack of capacity to perform both (13). PC role definition was critical for clinicians, staff and the public (31). Community PCPs in West Africa following EVD recommended formal recognition and funding of traditional birth attendants and community health providers, as well as clarifying their role in planning, implementing and monitoring community health initiatives (19).

##### Ensure that PHC clinicians are aware of pandemic preparedness plans

The need to consider pandemic preparedness plan implementation was emphasized, including formalized protocols and explicit mechanisms for distributing information and supplies to all PCPs, including ambulance paramedics (33). Studies across epidemics recommended that clinicians become aware of national pandemic plans to enable conformity (15,17,19,29). A survey of Japanese PCPs found an association between having read the national preparedness plan and establishment of a business continuity plan (17).

##### Provide preparedness plans and infection prevention and control training tailored to PHC

During the SARS outbreak, family doctors in Ontario reported not having the appropriate knowledge and skills to protect themselves, their patients, staff and their families (34,36); cancellation of medical education events increased their sense of professional isolation (34). Hong Kong PCPs reported having no PC-specific guidelines and infection prevention and control (IPC) procedures were not universally practiced (12). Surveys following SARS and H1N1 influenza found that the majority of PCPs had either no (11) or insufficient (28) training in infectious disease control, lacked confidence and required education to inform disease management (11,28). Recommendations included the provision of timely and relevant information regarding infectious disease outbreaks, use of personal protective equipment (PPE) (26,34) and incorporating IPC and PH training for PCPs and aged care staff (12,43) in ongoing clinical education programs (11).

##### Clarify pathways of care for suspected and confirmed cases, and maintain access to regular care

The need to clarify pathways of care (13,15,18,19,21,25,27,28,38,39,41) and maintain access to regular health care (13,15,16,19,20,38,41) was emphasized in studies examining SARS (25,27), H1N1 (13,15,16,28,38,39) and EVD (18–21,41). Australian PCPs described difficulties maintaining routine care, plus extended waiting times during H1N1 due to the increased volume of potential influenza patients (13). Recommendations included segregating care of affected and non-affected patients to maintain regular health care in parallel with care for those potentially infected (13,15,38), the use of centralized assessment centres (25) and screening facilities-based away from family practice clinics (27).

The need to consider the consequences of an epidemic on all aspects of care to avoid loss of services and unwarranted morbidity and mortality was identified after the African EVD epidemic (18,21). Fear of interacting with PCPs and outsiders led to a decline in maternal, newborn and child health activities, initiating recommendations to implement measures to mitigate poor outcomes for babies, mothers and families (18–20,32). A lack of trusted sources of community-engaged PHC was held responsible for the deaths of both infected and uninfected people, who were reluctant to seek treatment (32,41).

#### Protect the primary care workforce and the community

Nineteen studies from SARS (12,25–27,30,33–36), H1N1 (14,16,17,28,31,37–39), MERS (22) and EVD (19,32) identified the need to protect the PHC workforce.

##### Ensure access to antiviral treatments and vaccines, and protect priority groups

Identification and protection of staff and patients at greater risk of a poor outcome from infectious disease was recommended (38), including pregnant women (39). However, a cross-sectional survey of Hong Kong PCPs after the H1N1 influenza outbreak revealed that half were reluctant to have the influenza vaccine, prompting recommendations for interventions to increase uptake (28).

##### Promote health protective behaviours, psychological well-being and business continuity

During the SARS outbreak, PCPs in Singapore (26,27), Hong Kong (12) and Toronto (30,34,36) reported high levels of anxiety and fear for their own health, and of transmitting the virus to their families and others. For this reason, some chose not to provide care to affected patients during the H1N1 outbreak in Canada (16). Singaporean PCPs reported psychological distress related to caring for SARS patients, as well as stigma and post-traumatic stress (27).

During SARS, many PCPs in Hong Kong delayed or avoided quarantining themselves or staff until an infectious state was confirmed, perceived to be due to fear of lost income, especially for solo practitioners (12). Centrally organized contingency plans to mitigate business risks, and the provision of financial support for PCPs were recommended (12). Establishment of cohesive teams before the crisis, and clear documentation and communication systems supported adaptation by community pharmacists during SARS (30).

The provision of relevant information and training, and associated use of PPE, was considered one way to alleviate stress (26,36). Other recommendations included availability of prompt and accurate guidelines (12,27,28,36), immunizations (16), workload relief (12) and psychological support (12,27).

##### Provide personal protective equipment

Publications about SARS in Canada (36), Singapore (26,27), Canada (33,34) and Australia (25), and H1N1 in England (23), Australia (13,14,31,38,39) and Hong Kong (28) called for the adequate provision and funding of PPE, and support for staff training and compliance. PCPs and residential aged care facility staff described having inadequate amounts of the required equipment, especially PPE, to operate safely during SARS (12,26,27,33–35). Inadequate supplies of, and access to, PPE were also reported by PCPs during H1N1 (13,14,17,23,31,37–39). PCPs called for transparency in terms of the size of the national PPE stockpile, the distribution process and improved mechanisms to ensure ready and rapid release to PC facilities (14,37).

#### Evaluate the effectiveness of interventions

Rigorous assessment of the disease response, including training and supervision of health workers, accessibility and medication supplies was recommended (41), as was the need for clinical audits to evaluate the structure, process and outcomes of PC action plans (38). 

## Discussion

This review describes PC experiences during four infectious disease outbreaks (SARS, H1N1, MERS and EVD) in 11 countries over 14 years. Six key lessons emerged from these accounts. Epidemics and pandemics demand rapid system response to a new and uncertain clinical and epidemiological context, with a potentially high risk of morbidity and mortality of PCPs and their patients (44). In these circumstances, system weaknesses will be revealed, and the same weaknesses appear to have prevailed across outbreaks, time and, geographic and cultural boundaries. Consistently, these lessons resonate with issues currently emerging in the international discourse in response to COVID-19 (45).

### Improve collaboration, communication and integration between public health and primary care

The challenge of integrating PH, PC and the broader health system has been exposed during COVID-19 (46), and failure to include PC within pandemic planning has again been highlighted. Analysis of the composition of COVID-19 taskforces in 24 countries revealed a predominance of politicians, epidemiologists and virologists; with a notable absence of specialists in PC and other health and non-health matters who could provide relevant expertise regarding COVID-19 impacts on the social, emotional, economic and cultural well-being of the whole community (47). In contrast, some countries have endeavoured to ensure PC input to the decision-making process (48) and develop coordinated, whole-of-system responses to COVID-19. Despite this, we acknowledge that COVID-19 is pushing health services in many countries beyond capacity, at unprecedented levels, inevitably highlighting strengths and exposing system weaknesses (49). COVID-19 requires a coalition of representatives to consider a diversity of values and the varied impacts of any one potential solution (50).

While most studies identified the need for improved collaboration and communication between governments, PH and PC, two studies (16,31) identified barriers to collaboration that need to be considered in addressing this critical need. Pandemics are high-stake events, in which frontline workers can feel they are being placed at risk by decisions made by others, and PH workers are required to manage often exhausting and unappreciated burdens of responsibility in situations of uncertainty. Both studies noted that PH and PC practitioners often had limited understanding of the challenges faced by the other. The boundary-spanning roles of PCPs who have PH expertise, and vice versa, are critical in a pandemic to ensure integrated and mutually supportive service delivery.

### Strengthen the primary health care system

The need to strengthen health systems, particularly PC, and the importance of PC in supporting each nation’s pandemic response has been exemplified as the world grapples with COVID-19 (51,52). Reports of the burden on PCPs (53), reinforce the fact that strong PC underpins any effective health system response. Despite lessons learnt from previous epidemics, COVID-19 has exposed the same health system weaknesses in relation to disease detection and surveillance (49), prompting a call to consider centralized coordination of surveillance and infectious disease response (54). Such a system has been implemented in England; however, inadequate information sharing and communication delays have slowed the responses of general practice and PH teams which, at local and regional levels, are best placed to understand the needs of the communities they serve (55).



##### Provide consistent, coordinated and reliable information emanating from a trusted source

While it is not unusual for experts to have differing opinions during a pandemic, the need for consistent, coordinated and reliable information, emanating from trusted sources, was resounding in the papers included in our review. The importance of consistent and trustworthy advice has been emphasized again during COVID-19 (56). English PCPs reported information coming from a variety of sources, at times conflicting and not PC-specific (57), and a lack of information sharing has hampered PC interventions (55).

### Define the role of primary care during pandemics

This review suggests that the PC role could be clarified and strengthened by each nation, recognising and supporting PCPs’ dual roles in providing clinical care and supporting PH control measures. A synthesis of PCPs’ experiences in 68 countries reported that PCPs in Singapore, Taiwan, Hong Kong and South Korea were well-prepared as a result of lessons learnt from SARS and MERS epidemics; however, PCPs in all other countries reviewed were ill-prepared and felt ill-informed of how to fulfil their roles (53). Despite this, PCPs demonstrated agility, resilience and creativity in their responses through segregating care pathways (53,57), optimizing digital access to care (53) and sharing workforces (57).

### Clarifying pathways of care

Our review emphasizes the critical importance of clarifying pathways of care for preventing disease transmission and ensuring safe and continued access to regular health care. International health system responses to COVID-19 have worked to maintain access to regular health care (48,58), including enhanced access to telehealth (48,53,57,59), and establishment of dedicated respiratory assessment, COVID-19 testing and treatment clinics (53). However, telehealth has its limitations, particularly for those without smartphones or with limited network connectedness (57). At the same time, measures aimed at controlling COVID-19, such as social and physical isolation, have resulted in access delays to important and urgent care (53), vaccinations (60–62), infectious (61,63) and chronic disease treatments (61,64,65), mental health (61) and maternal and child health programs (61,66).

### Protect the primary care workforce and the community

Although only one study in our review highlighted the importance of identifying and protecting vulnerable individuals, inequity and social disadvantage contribute to both infectious disease spread and amplification of the effects of a pandemic (67). Early reports during the COVID-19 pandemic refer to unintended consequences of social isolation measures, including diminished access to food (68), and increased psychological distress for those with mental health needs (69) and disabilities (70). Protection of vulnerable individuals has been a feature of some countries’ responses to COVID-19 (45).

Protecting the health care workforce is critical during a pandemic. Despite relative early success in suppressing COVID-19 transmission, approximately 15% of cases in Victoria, Australia in August 2020 were health care workers (71). Isolating health care workers who have been exposed to COVID-19 can have a detrimental effect on the capacity to manage health care demands, and the risk of disease transmission related to staff attending work when ill is substantial (72).

Studies included in this review revealed high levels psychological distress and fear in PCPs related to fear of exposure to the infectious agent and of transmitting the infection to others; lack of access to appropriate PPE; lack of PC-relevant training and guidelines impacting safe work practices; and lack of clear and consistent information from reliable sources. Unfortunately, similar issues have arisen among PCPs during COVID-19, particularly related to insomnia, anxiety and depression (73), loss of income (74), fear of contracting COVID-19 and passing it on to others, and access to PPE (75,76).

### Evaluate the effectiveness of interventions

While only two studies included in our review referred to the need for evaluation of pandemic responses, we believe the evaluation is critical. Noting that many lessons from the past have re-emerged during COVID-19, the need to embed research, evaluation and continuous quality improvement into PHC efforts can accelerate progress and provide real-time feedback and guidance to inform policy (51). Continuous dialogue between policy-makers and researchers strengthens learnings, and can embed a sense of ownership and legitimacy of research and evaluation by the health system and policy-makers (77). This may be one way to shorten the knowledge translation gap and ensure that PHC lessons from pandemics, such as COVID-19, are embedded in ongoing and future pandemic responses.

Strategies that can be developed to address the six key lesson identified in this review are mostly underpinned by the need to improved integration of PC and PH functions. Provision of consistent, coordinated and reliable information, clarifying PC roles and protection of vulnerable people, including health care workers, are all dependent on a critical starting point of effective collaboration and joint preparedness planning between PC and PH agencies. However, this is not without challenges and ambitious strategies such as the real-time extraction of sentinel data are dependent on factors such as individual provider and practice capability, and may not be feasible across all health systems.

## Limitations

A focus of our review has been on the interaction between PC and PH as components of PHC, a lens which necessarily removes the focus from other parts of the health system and other sectors. A broader perspective on PHC might also identify intersectoral issues arising from infectious disease outbreaks, inclusive of the education, transport, labour market and food sectors, referred to as intersectoral action for health (41). A system focus beyond PHC might incorporate hospitals and other care settings, and explore process and communication issues not identified in this review.

The search strategy may have excluded papers with lessons of relevance to PC that were framed from a PH perspective but did not explicitly refer to PC settings. Meta-analysis was not possible due to the qualitative nature of most included studies. The use of one reviewer to screen and extract data may have introduced bias into this review, which is a limitation and trade-off in rapid review methods.

We did not find a particular emphasis on health inequity and vulnerability in this review, although these issues have been a strong theme in the emerging literature on the impact of COVID-19 (78). People with vulnerabilities may be more likely to turn to PC practices and providers during a pandemic, where they provide services that are perceived as culturally or psychologically safe and familiar; a circumstance described in some reports we examined (36).

## Conclusion

Despite the harm caused and lessons documented from past epidemics, COVID-19 has exposed the same PHC health system weaknesses. Our review demonstrates the ongoing challenges of integrating PC and PH, the case for strengthening PC involvement in pandemic planning and response - with clear PC and PH role definition, the importance of providing clear and consistent information, and the importance of protecting the health care workforce and the community. The visible reminder of these weaknesses provides an opportunity for action; one that should not be ignored in a world where the frequency, virility and global reach of infectious disease outbreaks are increasing.

## Declaration

Funding: No funding to declare.

Data availability: The data underlying this article will be shared on a reasonable request to the corresponding author.

## References

[1] World Health Organisation T. *Severe Acute Respiratory Syndrome (SARS)*. 2020. https://www.who.int/health-topics/severe-acute-respiratory-syndrome#tab=tab_1 (accessed on 6 August 2020).

[2] Prevention CfDCa. *2009 H1N1 Pandemic (H1N1pdm09 Virus)*. 2020. https://www.cdc.gov/flu/pandemic-resources/2009-h1n1-pandemic.html (accessed on 16 July 2020).

[3] World Health Organisation T. *Middle East respiratory syndrome coronavirus (MERS-CoV).* 2020. https://www.who.int/health-topics/middle-east-respiratory-syndrome-coronavirus-mers#tab=tab_1 (accessed on 6 August 2020).

[4] Decroo T, Fitzpatrick G, Amone J. What was the effect of the West African Ebola outbreak on health programme performance, and did programmes recover? Public Health Action 2017; 7(Suppl 1): 1–2. 28744431 10.5588/pha.17.0029 PMC5515555

[5] Musso D, Ko AI, Baud D. Zika virus infection—after the pandemic. New Engl J Med 2019; 381(15): 1444–57. 31597021 10.1056/NEJMra1808246

[6] World Health Organisation T. Coronavirus disease (COVID-19) Situation Report – 178. Geneva: World Health Organisation, 2020.

[7] Rawaf S AL, Dubois E, Majeed A, et al Primary Health Care: Closing the Gap between Public Health and Primary Care through Integration. Geneva: The World Health Organisation, 2018.

[8] Haby MM, Chapman E, Clark R, Barreto J, Reveiz L, Lavis JN. What are the best methodologies for rapid reviews of the research evidence for evidence-informed decision making in health policy and practice: a rapid review. Health Res Policy Syst 2016; 14(1): 83. 27884208 10.1186/s12961-016-0155-7 PMC5123411

[9] Tricco AC, Antony J, Zarin W, et al A scoping review of rapid review methods. BMC Med 2015; 13(1): 224. 26377409 10.1186/s12916-015-0465-6 PMC4574114

[10] Levesque J-F, Breton M, Senn N, Levesque P, Bergeron P, Roy DA. The interaction of public health and primary care: functional roles and organizational models that bridge individual and population perspectives. Public Health Rev 2013; 35(1): 14.

[11] Wong WCW, Wong SYS, Lee A, Goggins WB. How to provide an effective primary health care in fighting against severe acute respiratory syndrome: the experiences of two cities. Am J Infect Control 2007; 35(1): 50–5. 17276791 10.1016/j.ajic.2006.06.009 PMC7132727

[12] Wong WCW, Lee A, Tsang KK, Wong SYS. How did general practitioners protect themselves, their family, and staff during the SARS epidemic in Hong Kong? J Epidemiol Community Health 2004; 58(3): 180–5. 14966227 10.1136/jech.2003.015594 PMC1732708

[13] Bocquet J, Winzenberg T, Shaw KA. Epicentre of influenza—the primary care experience in Melbourne, Victoria. Austr Fam Physician 2010; 39(5): 313–6. 20485719

[14] Eizenberg P. The general practice experience of the swine flu epidemic in Victoria—lessons from the front line. Med J Austr 2009; 191(3): 151–3. 10.5694/j.1326-5377.2009.tb02725.x 19645644

[15] Kunin M, Engelhard D, Thomas S, Ashworth M, Piterman L. Challenges of the pandemic response in primary care during pre-vaccination period: a qualitative study. ISR J Health Policy Res 2015; 4: 32. 26473026 10.1186/s13584-015-0028-5 PMC4606524

[16] Masotti P, Green ME, Birtwhistle R et al pH1N1—a comparative analysis of public health responses in Ontario to the influenza outbreak, public health and primary care: lessons learned and policy suggestions. BMC Public Health 2013; 13: 687. 23890226 10.1186/1471-2458-13-687 PMC3726397

[17] Tomizuka T, Kanatani Y, Kawahara K. Insufficient preparedness of primary care practices for pandemic influenza and the effect of a preparedness plan in Japan: a prefecture-wide cross-sectional study. BMC Fam Pract 2013; 14: 174. 24252688 10.1186/1471-2296-14-174 PMC3840630

[18] Iyengar P, Kerber K, Howe CJ, Dahn B. Services for mothers and newborns during the Ebola outbreak in Liberia: the need for improvement in emergencies. PLoS Currents 2015; 7. 10.1371/currents.outbreaks.4ba318308719ac86fbef91f8e56cb66f PMC4404271 25932347

[19] Miller NP, Milsom P, Johnson G, et al Community health workers during the Ebola outbreak in Guinea, Liberia, and Sierra Leone. J Global Health 2018; 8(2): 020601. 10.7189/jogh-08-020601 PMC6030670 30023054

[20] Siekmans K, Sohani S, Boima T, Koffa F, Basil L, Laaziz S. Community-based health care is an essential component of a resilient health system: evidence from Ebola outbreak in Liberia. BMC Public Health 2017; 17(1): 84. 28095824 10.1186/s12889-016-4012-y PMC5240441

[21] Wagenaar BH, Augusto O, Beste J et al The 2014-2015 Ebola virus disease outbreak and primary healthcare delivery in Liberia: time-series analyses for 2010−2016. PLoS Med 2018; 15(2): e1002508. 29462138 10.1371/journal.pmed.1002508 PMC5819774

[22] Al-Amri S, Bharti R, Alsaleem SA, Al-Musa HM, Chaudhary S, Al-Shaikh AA. Knowledge and practices of primary health care physicians regarding updated guidelines of MERS-CoV infection in Abha city. J Family Med Prim Care 2019; 8(2): 455–61. 30984654 10.4103/jfmpc.jfmpc_336_18 PMC6436268

[23] Caley M, Sidhu K, Shukla R. GPs’ opinions on the NHS and HPA response to the first wave of the influenza A/H1N1v pandemic. Br J Gen Pract 2010; 60(573): 283–5. 20353672 10.3399/bjgp10X483968 PMC2845489

[24] El Emam K, Mercer J, Moreau K, Grava-Gubins I, Buckeridge D, Jonker E. Physician privacy concerns when disclosing patient data for public health purposes during a pandemic influenza outbreak. BMC Public Health 2011; 11: 454. 21658256 10.1186/1471-2458-11-454 PMC3130674

[25] Herceg A, Geysen A, Guest C, Bialkowski R. SARS and biothreat preparedness—a survey of ACT general practitioners. Commun Dis Intell Q Rep 2005; 29(3): 277–82. 16220864 10.33321/cdi.2005.29.27

[26] Tan NC, Goh LG, Lee SS. Family physicians’ experiences, behaviour, and use of personal protection equipment during the SARS outbreak in Singapore: do they fit the Becker Health Belief Model? Asia Pac J Public Health 2006; 18(3): 49–56. 10.1177/10105395060180030901 17153082

[27] Verma S, Mythily S, Chan YH, Deslypere JP, Teo EK, Chong SA. Post-SARS psychological morbidity and stigma among general practitioners and traditional Chinese medicine practitioners in Singapore. Ann Acad Med Singap 2004; 33(6): 743–8. 15608831

[28] Wong SY, Kung K, Wong MC et al Primary care physicians’ response to pandemic influenza in Hong Kong: a mixed quantitative and qualitative study. Int J Infect Dis 2012; 16(9): e687–91. 22789752 10.1016/j.ijid.2012.03.015 PMC7128972

[29] Kunin MMAP, Engelhard DMD, Thomas SPM, Ashworth MDM, Piterman LAM. Influenza pandemic 2009/A/H1N1 management policies in primary care: a comparative analysis of three countries. Austr Health Rev 2013; 37(3): 291–9. 10.1071/AH12022 23731961

[30] Austin Z, Martin JC, Gregory PA. Pharmacy practice in times of civil crisis: The experience of SARS and the blackout in Ontario, Canada. Res Social Adm Pharm 2007; 3(3): 320–35. 17945161 10.1016/j.sapharm.2006.09.001 PMC7106290

[31] Phillips C. Resistance and Accommodation in Pandemic Preparation and Response in General Practice and the Ethnographic Eye: Culture, Change and the Organisation: Canberra, Australia: Australian National University; 2016.

[32] National Academy of Medicine. Commission on a Global Health Risk Framework for the Future. The Neglected Dimension of Global Security: A Framework to Counter Infectious Disease Crises. Washington (DC): National Academies Press (US) Copyright 2016 by the Commission on a Global Health Risk Framework for the Future, 2016. 27336117

[33] National Advisory Committee on SARS and Public Health. *Learning from SARS: Renewal of Public Health in Canada*. Ottawa: Health Canada, 2003.

[34] Ontario College of Family Physicians. *The Mushroom Syndrome: SARS and Family Medicine*. Toronto: OCFP, 2003.

[35] SARS Expert Committee. *SARS in Hong Kong: from Experience to Action*. Hong Kong: The Government of Hong Kong, 2003.

[36] SARS Commission T. Spring of Fear. *Commission to Investigate the Introduction and Spread of SARS in Ontario*, 2006. http://www.archives.gov.on.ca/en/e_records/sars/report/index.html.

[37] Grayson ML, Johnson PDR. Australia’s influenza containment plan and the swine flu epidemic in Victoria. Med J Austr 2009; 191(3): 150–. 10.5694/j.1326-5377.2009.tb02724.x 19645643

[38] Lee A, Chuh AA. Facing the threat of influenza pandemic—roles of and implications to general practitioners. BMC Public Health 2010; 10: 661. 21044300 10.1186/1471-2458-10-661 PMC2988738

[39] Sweet M. Pandemic lessons from Australia. BMJ: Br Med J (Online) 2009; 339: b3317. 10.1136/bmj.b3317 19690003

[40] Al Shehri AM. A lesson learned from Middle East respiratory syndrome (MERS) in Saudi Arabia. Med Teacher 2015; 37: S88–93. 10.3109/0142159X.2015.1006610 25803593

[41] Scott V, Crawford-Browne S, Sanders D. Critiquing the response to the Ebola epidemic through a Primary Health Care Approach. BMC Public Health 2016; 16: 410. 27185252 10.1186/s12889-016-3071-4 PMC4869325

[42] Kunin M, Engelhard D, Piterman L, Thomas S. Response of general practitioners to infectious disease public health crises: An integrative systematic review of the literature. Disaster Med Public Health Preparedness 2013; 7(5): 522–33. 10.1017/dmp.2013.82 24274132

[43] Committee SE. SARS in Hong Kong: from Experience to action. Hong Kong: HK Special Administrative Region, 2003.

[44] Pham T-N, Powis J, Fam M, Fraser I, Wojtak A. Early lessons: tackling a global crisis with a community response. Insights (essays). Longwoods, 2020. https://www.longwoods.com/content/26167/essays/early-lessons-tackling-a-global-crisis-with-a-community-response.

[45] Kidd M. Principles for primary care pandemic preparedness: lessons from the Australian COVID-19 primary care response. Br J Gen Pract 2020; 70 (696): 316–317. 32571772 10.3399/bjgp20X710765 PMC7311106

[46] Cheney C. *New IHI Chief Executive: ‘There is no quality without equity’*. July 8, 2020. https://www.healthleadersmedia.com/clinical-care/new-ihi-chief-executive-there-no-quality-without-equity (accessed on 15 July 2020).

[47] Rajan D, Koch K, Rohrer K, et al Governance of the Covid-19 response: a call for more inclusive and transparent decision-making. BMJ Global Health 2020; 5: e002655. 10.1136/bmjgh-2020-002655 PMC7228498 32371570

[48] Desborough J, Hall Dykgraaf S, de Toca L et al Australia’s national COVID-19 primary care response. Med J Aust 2020; 213(3): 104–106.e1. 32623740 10.5694/mja2.50693 PMC7361540

[49] Craven M, Sabow A, Van der Veken L, Wilson M. *Not the Last Pandemic: Investing Now to Reimagine Public-Health Systems*. 2020. https://www.mckinsey.com/industries/public-sector/our-insights/not-the-last-pandemic-investing-now-to-reimagine-public-health-systems?cid=other-onw-onw-mip-mck-oth-2007&hlkid=8869ec6090574c0c881ddce7784c1842&hctky=11320267&hdpid=c8f507dc-ae1a-4618-b360-b136a1a9dfa1# (accessed on 15 July 2020).

[50] Patel MS, Phillips C. COVID, the wicked problem too big for medical experts alone to tackle. The Sydney Morning Herald. 2020 July 25, 2020.

[51] World Health Organisation. *Embedded Primary Health Care Research to Engage Communities and Build Learning Health Systems (Focus: COVID-19 and Emergency Preparedness)*. 2020. https://iris.wpro.who.int/handle/10665.1/14620 (accessed on 14 July 2020). https://iris.wpro.who.int/handle/10665.1/14620

[52] Dunlop C, Howe A, Li D, Allen LN. The coronavirus outbreak: the central role of primary care in emergency preparedness and response. BJGP Open 2020; 4(1): bjgpopen20X101041. 10.3399/bjgpopen20X101041 PMC7330191 31992543

[53] Rawaf S, Allen LN, Stigler FL et al. Lessons on the COVID-19 pandemic, for and by primary care professionals worldwide Eur J Gen Pract 2020; 26(1): 129–33. 32985278 10.1080/13814788.2020.1820479 PMC7534357

[54] Cheng AC, Williamson DA. An outbreak of COVID‐19 caused by a new coronavirus: what we know so far. Med J Austr 2020; 212(9): 393–4e1. 10.5694/mja2.50530 PMC7228293 32146721

[55] Nazareth J, Minhas JS, Jenkins DR et al Early lessons from a second COVID-19 lockdown in Leicester, UK. Lancet 2020; 396(10245): e4–5. 32622374 10.1016/S0140-6736(20)31490-2 PMC7330565

[56] Desborough J, Hall Dykgraaf S, Rankin D, Kidd M. Importance of consistent advice during a pandemic. Austr J Gen Pract 2020; 49: 369–72. 10.31128/AJGP-04-20-5374 32464735

[57] Thornton J. Covid-19: how coronavirus will change the face of general practice forever. BMJ 2020; 368: m1279. 32229477 10.1136/bmj.m1279

[58] Lim WH, Wong WM. COVID-19: Notes from the front line, Singapore’s primary health care perspective. Ann Fam Med 2020; 18(3): 259–61. 32393562 10.1370/afm.2539 PMC7214001

[59] Mahal I. Coronavirus has sped up Canada’s adoption of telemedicine. Let’s make that change permanent. The Conversation 2020; **April 5**, 2020: https://theconversation.com/coronavirus-has-sped-up-canadas-adoption-of-telemedicine-lets-make-that-change-permanent-134985.

[60] Farmer B. Pakistan to resume polio vaccination campaign months after it was halted by coronavirus. The Telegraph. 2020.

[61] World Health Organisation. *Pulse Survey on Continuity of Essential Health Services during the COVID-19 Pandemic: Interim Report, 27 August 2020*. Geneva: WHO, 2020.

[62] Newey S. Measles and polio may come ‘roaring back’ as global vaccination programmes shut down. The Telegraph. 2020 31 March 2020.

[63] Cilloni L, Fu H, Vesga JF, et al The potential impact of the COVID-19 pandemic on tuberculosis: a modelling analysis. EClinicalMedicine 2020; 28: 100603. 33134905 10.1016/j.eclinm.2020.100603 PMC7584493

[64] Ghosal S, Sinha B, Majumder M, Misra A. Estimation of effects of nationwide lockdown for containing coronavirus infection on worsening of glycosylated haemoglobin and increase in diabetes-related complications: a simulation model using multivariate regression analysis. Diabetes Metab Syndr 2020; 14(4): 319–23. 32298984 10.1016/j.dsx.2020.03.014 PMC7146694

[65] Wu T. Diabetes patients at risk after lockdown neglect. AusDoc Plus 2020; **June 5,** 2020: https://www.ausdoc.com.au/sponsored/diabetes-patients-risk-after-lockdown-neglect.

[66] Poudel A. A 200 percent increase in maternal mortality since the lockdown began. The Kathmandu Post 2020; **May 27,** 2020: https://kathmandupost.com/national/2020/05/27/a-200-percent-increase-in-maternal-mortality-since-the-lockdown-began.

[67] Quinn SC, Kumar S. Health inequalities and infectious disease epidemics: a challenge for global health security. Biosecur Bioterror 2014; 12(5): 263–73. 25254915 10.1089/bsp.2014.0032 PMC4170985

[68] Cash R, Patel V. Has COVID-19 subverted global health? Lancet 2020; 395(10238): 1687–8. 32539939 10.1016/S0140-6736(20)31089-8 PMC7200122

[69] Lee J. Mental health effects of school closures during COVID-19. The Lancet Child Adolescent Health 2020; 4(6): 421. 32302537 10.1016/S2352-4642(20)30109-7 PMC7156240

[70] Tuffrey-Wijne I. Professor of intellectual disability and palliative care. http://www.tuffrey-wijne.com/?p=840 (accessed on 18 May 2020).

[71] Scott S, Lloyd M, Clark E. *Healthcare Workers Make Up More Than15% of Victoria’s New Coronavirus Cases*. 2020. https://www.abc.net.au/news/2020-08-11/doctors-warn-of-coronavirus-in-victorian-healthcare-workers/12544884 (accessed on 18 August 2020).

[72] Hall Dykgraaf S, Desborough J, Kelaher C, Kidd M. COVID 19 highlights risks of healthcare and social care workers attending work while ill. Austr J Gen Pract 2020; 49 (Suppl 23). doi:10.31128/AJGP-COVID-23 32539245

[73] Pappa S, Ntella V, Giannakas T, Giannakoulis VG, Papoutsi E, Katsaounou P. Prevalence of depression, anxiety, and insomnia among healthcare workers during the COVID-19 pandemic: a systematic review and meta-analysis. Brain Behav Immunity 2020 88: 901–7. 10.1016/j.bbi.2020.05.026 PMC7206431 32437915

[74] Scholefield A. *GPs Say Income is Down Significantly with Some Fearing for the Future: RACGP Survey*. 2020. https://www.ausdoc.com.au/news/gps-say-income-down-significantly-some-fearing-future-racgp-survey?mkt_tok=eyJpIjoiT1Rrek9XTm1aR013WWpCaCIsInQiOiJRd2ROSjVJNXI0OFdJYkR6U1djUGhkZHdnUDZZbGtSTlptQ1NyMGhaMlVmSmNoUGlPUFZhczBNWExjNlZrYmtVRUpiMkR2VHkxNDdiOEppMHJhM09tbEtcL3psTlVVQmNGd1RGZUthTHFram9VTzZHNUJUdXUxQURhMnN2bGtqRmwifQ%3D%3D (accessed on 21 June 2020).

[75] Pfefferbaum B, North CS. Mental Health and the Covid-19 Pandemic. N Engl J Med 2020; 383(6): 510–2. 32283003 10.1056/NEJMp2008017

[76] Thielking M. Frustrated and afraid about protective gear shortages, health workers are scouring for masks on their own. Stat. 2020 18 Mar 2020.

[77] Ghaffar A, Langlois EV, Rasanathan K, Peterson S, Adedokun L, Tran NT. *Strengthening Health Systems through Embedded Research*. 2017. https://www.who.int/bulletin/volumes/95/2/16-189126/en/ (accessed on 17 July 2020). 10.2471/BLT.16.189126 PMC5327943 28250505

[78] Shadmi E, Chen Y, Dourado I et al Health equity and COVID-19: global perspectives. Int J Equity Health 2020; 19(1): 104. 32586388 10.1186/s12939-020-01218-z PMC7316580

